# Lower risk of postoperative complications with Hardinge approach in revision total hip replacement surgery

**DOI:** 10.1051/sicotj/2026011

**Published:** 2026-10-21

**Authors:** Seng Juong Wong, Sasidaran Ramalingam, Andy Yew Khye Soon, Joel Yat Seng Wong, Audrey Xinyun Han, Eric Xuan Liu, Lincoln Ming Han Liow, Darren Keng Jin Tay, Seng Jin Yeo, Hee Nee Pang

**Affiliations:** 1 Singapore General Hospital 10 Hospital Boulevard Singapore 168582 Singapore; 2 Hopsital Tuanku Ja’afar, Jalan Rasah, Bukit Rasah 70300 Seremban Negeri Sembilan Malaysia

**Keywords:** Revision total hip replacement, Hardinge approach, Posterior approach, Dislocation rate, Patient-reported outcomes (PROMs)

## Abstract

*Introduction*: The increasing volume of total hip replacement (THR), coupled with longer life expectancy and greater patient demands, is expected to result in a corresponding increase in revision THR. The surgical approach used for revision THR may influence postoperative outcomes, particularly the risk of dislocation. This study aimed to compare clinical outcomes following revision THR performed through the lateral and posterior approaches, with particular emphasis on postoperative dislocation rates. *Methods*: We retrospectively reviewed prospectively collected registry data from 137 patients who underwent revision THR. Of these, 81 underwent revision THR through a lateral approach and 56 through a posterior approach. Propensity scores were generated using logistic regression to adjust for potential confounders, including age, sex, body mass index, and preoperative patient-reported outcome measures (Oxford Hip Score, WOMAC Pain, Stiffness and Physical Function scores, and SF-36 Physical and Mental Component Scores). Postoperative outcomes, including dislocation, blood loss, patient-reported outcome measures, and satisfaction, were compared between groups. *Results*: After propensity score matching, the incidence of dislocation was significantly higher in the posterior approach group than in the lateral approach group (16.1% vs. 1.8%, *p* = 0.02). The lateral approach was also associated with significantly lower postoperative blood loss compared with the posterior approach (change in haemoglobin: 1.5 vs. 2.5 g/dL, p < 0.001). No significant differences were observed between groups in SF-36, WOMAC, or Oxford Hip Scores at 6 months or 2 years postoperatively. However, patient satisfaction was significantly higher following the lateral approach at both 6 months (87.5% vs. 73.2% satisfied or very satisfied, *p* = 0.02) and 2 years (87.5% vs. 67.9%, *p* = 0.019). *Discussion*: In this cohort, revision THR performed through a lateral approach was associated with a significantly lower dislocation rate, lower blood loss, and higher patient satisfaction compared with the posterior approach, without significant differences in functional or health-related quality-of-life outcomes. The lateral approach appears to be a viable option for revision THR, particularly when minimising postoperative instability is a priority. Further comparative studies are warranted to validate these findings and determine the influence of surgical approach on outcomes following revision THR.

## Introduction

Total hip replacement (THR) is often regarded as the surgery of the century due to its favourable clinical and functional outcomes and excellent survivorship [1]. As a result, an increasing number of THR procedures are being performed, with improving patients’ quality of life being a primary goal [2, 3]. However, it is expected that the number of revision surgeries will increase in tandem, given the increasing life expectancy and demand among patients [4].

The mean rate of revision for any reason following total hip replacement surgery is 1.29 revisions per 100 observed component years, based on data from six national joint registries [5]. This corresponds to a revision rate of 6.45% after five years and 12.9% after ten years. The data comes from a cumulative total of 689,608 primary operations and 79,231 revision operations, with an average follow-up period of 8.91 years [5].

Causes for revision of total hip replacement (THR) can be classified based on time of occurrence: early and late. In late failures, aseptic loosening was the primary reason for revision, while in early failures, the most significant causes were instability and deep infection. These early failures were often directly related to errors in surgical technique, such as component malposition, soft tissue laxity, or bacterial contamination of the operative field, resulting in prosthetic joint infection [6].

Currently, posterior, lateral, and anterior approaches are the three main surgical approaches for total hip replacement (THR). The posterior approach is preferred by many surgeons due to its reduced tissue dissection, shorter operating times, and lower blood loss. It also provides excellent femoral exposure while minimizing the risk of femoral fracture during the procedure. Additionally, this method avoids damage to the abductor muscles, thereby reducing postoperative gait problems. However, it may impede acetabular visualization and increase the risk of posterior dislocation, which may require additional measures to mitigate this risk [7].

The direct lateral approach offers several advantages, including improved visualization of the acetabulum, which facilitates optimal placement of the acetabular cup and potentially reduces the incidence of hip dislocation. Additionally, this approach minimizes the risk of sciatic nerve injury as it is located posterior to the surgical field. However, there is a higher risk of superior gluteal nerve injury and associated gluteus medius muscle damage, which may result in a Trendelenburg gait pattern [8].

Postoperative THR dislocation is a dreaded complication due to its high risk of morbidity and cost. The incidence of dislocation following primary total hip replacement (THR) ranges from 0.5% to 6%, while the incidence of dislocation following revision THR can go up to as high as 10% and is one of the most common causes of failure in revision THR. This phenomenon does not appear to decrease with increasing surgeon experience [9]. To our knowledge, no one has evaluated the risk of dislocation in revision THR, and this has the potential to drastically decrease this significant complication.

The aim of this study is to compare the dislocation rates of patients undergoing revision total hip replacement via the lateral and posterior approach. Secondary outcomes, looking at postoperative complications and functional outcomes, are compared.

## Methods

An IRB was obtained to study outcomes of all total hip replacements performed at our institution, which included outcomes of revision total hip replacements (CIRB Ref: 2018/2386). Written informed consent was obtained from all patients and/or families.

All patients undergoing revision THR surgeries from 2012 to 2022 were identified from our adult reconstruction unit registry data. Revision surgeries were performed by fellowship-trained arthroplasty surgeons, and data were collected prospectively by blinded physiotherapists on various variables including age, body mass index (BMI), reasons for revision surgery, pre- and postoperative hemoglobin values, radiological findings, and functional outcomes.

The study included a total of 81 patients who underwent the lateral approach and 56 patients who underwent the posterior approach, and both groups matched through propensity matching based on age, BMI, gender, and diagnosis. All index primary hip replacements were performed using the posterior approach.

The inclusion criteria for this study were revision THR surgeries with either a lateral or a posterior approach in the revision setting. Primary complex THR surgeries, THR following removal of internal fixators, anterior approach procedures, and resection arthroplasty cases were excluded. All implants used for our series of revision THR were Zimmer Biomet trabecular metal cup and Wagner stems with either a 32 or 36 mm head, with no significant differences between the lateral and posterior group (*p* = 0.252). In the lateral group (*n* = 81), there were 75 cases with a 36 mm head, and 6 cases of 32 mm head, whereas in the posterior group(*n* = 56), there were 49 cases of 36 mm head and 7 cases of 32 mm head (Figure 1).

**Figure 1:**
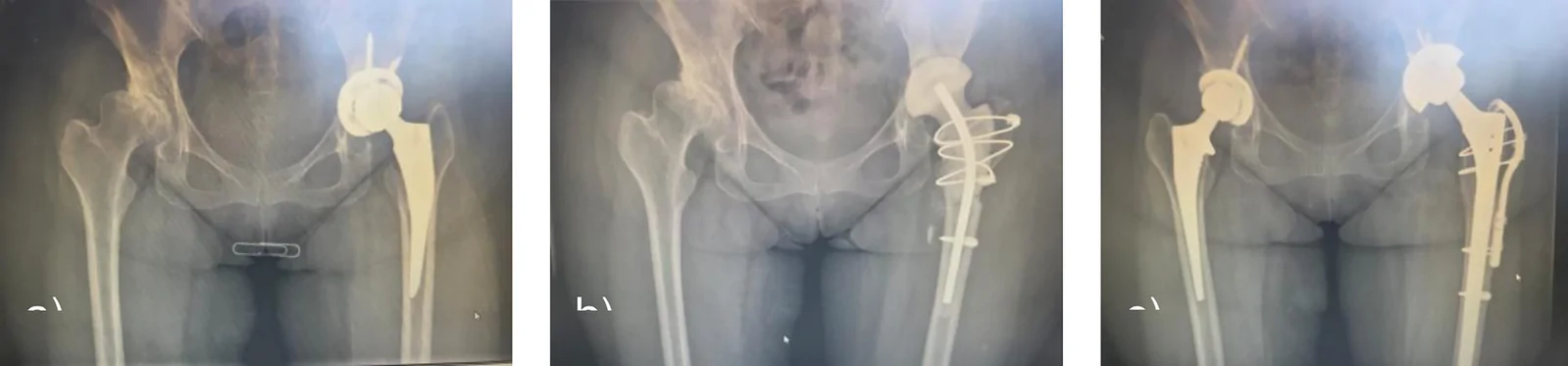
(a)–(c): Highlighting a case of 67/F with left total hip replacement done for hip dysplasia with subsequent prosthetic joint infection that underwent two-stage revision. A lateral approach was employed which allowed extended trochanteric osteotomy to be performed and subsequent greater trochanteric claw plate to be used. Stability was achieved with a 52 mm trabecular metal cup and a 32 mm head. Prophylactic cables were placed distally.

The lateral approach utilized in our patient cohort represents a slight variation of the original Hardinge technique [10]. A lateral longitudinal incision was made over the greater trochanter. The iliotibial band was incised, and the bursa was excised to expose the gluteus medius. The anterior and posterior margins of the gluteus medius are then identified, and the anterior one-third of the abductor is carefully released in an Omega curve shape, while ensuring that a cuff of the abductor remains attached to the greater trochanter for future repair. This approach is further extended distally into the vastus lateralis. In the proximal deeper plane, the gluteus minimus and capsule were incised together to expose the hip joint. This approach was continued distally to release the vastus lateralis off the proximal femur. Since the index surgery’s approach was posterior, the tissue planes in this modified Hardinge approach were easily identifiable, and repair of the capsule was possible in all cases.

Posterior approach used in revision setting is a well-described approach, starting with a curvilinear incision over the gluteal region using the greater trochanter and posterior superior iliac spine as landmarks [11]. The gluteus maximus was incised, and the entire posterior soft tissue envelope (short external rotators and capsule) was released from the proximal femur. Upon closure, a capsular repair was performed with short external rotators reattached to the greater trochanter using a trans osseous approach. During posterior approach revision THR, soft tissue dissection comprised of going through the same incision made in the index surgery, extended if necessary, and releasing the previous short external rotators repair and capsulotomy to reach the hip joint. During closure, deep soft tissues are closed to prevent dislocation.

Functional outcome details collected in this study are the Oxford Hip Score, WOMAC Score, and SF-36 scoring [12]. These scores were collected prospectively by physiotherapists who were blinded to the nature of the study. The analysis was conducted at intervals of 6 months, 1 year, and 2 years postoperatively by the Orthopaedic Diagnostic Centre at Singapore General Hospital. Radiological measurements were taken using a PACS system to measure the postoperative positioning of the acetabular cup, and these measurements were conducted by three senior surgeons, and an average of their measurements was taken to minimize the risk of inter-observer errors.

A total of 137 patients with revision total hip replacement (THR) were included. From this dataset, 81 lateral approach and 56 posterior approach revision THR were identified.

Testing of normality was performed on these two groups of revision THR using the Shapiro-Wilk test. Comparison of the demographics and outcome scores was performed next. Continuous variables with normal distribution were analysed with Student's t-test, while non-parametric data were analysed with the Mann-Whitney U test. Chi-square test was used to analyze categorical variables.

Because of the uneven number of patients in the two groups, propensity score matching (PSM) was next performed on these 137 patients with revision THR. The effects of patients with lateral approach THR versus patients with posterior approach THR were investigated. Propensity scores were generated using logistic regression to adjust for confounding variables of age, sex, BMI, and pre-treatment scores (Oxford, WOMAC: Pain, Stiffness, Physical Function, SF36: Physical and Mental Component Scores). Each patient in the treatment group was matched to another patient in the non-treatment group with the closest propensity score using the nearest-neighbor method and a 1:1 matching without replacement. After matching, comparison of the two groups was again conducted.

Data analysis was performed using R (Version 4.02) [R core Team, 2015], and statistical significance was defined as a *P*-value of less than 0.05.

## Results

### Patient demographics

Statistically significant differences were observed in the lateral group versus posterior group before and after propensity score matching for BMI (24.2 vs. 27, *p* = .007 and 24.4 vs. 27, *p* = 0.027), and blood loss measured as drop in hemoglobin (1.6 g/dL vs. 2.5 g/dL, *p* = .001 and 1.5 g/dL vs. 2.5 g/dL, *p* < 0.001). There is no statistical difference in terms of age after matching analysis (70 vs. 66.1, *p* = 0.058). Cause for THR revision was also statistically significantly different (before propensity score matching, *p* < .001 and after matching, *p* = 0.017) with a higher number of aseptic loosening and dislocation observed in the posterior group as compared with the lateral group.

### Dislocation rates

Significantly higher incidence of dislocation after revision THR was noted in the posterior group (16.1%) compared to the lateral group (1.2%) (*p* = 0.003) before propensity score matching. After matching, similar findings continued to be observed with a higher incidence of dislocation after revision THR in the posterior group compared to the lateral group (16.1% vs. 1.8%, *p* = 0.02).

### Blood loss and transfusion requirement

Lateral approach revision THR results in significantly lower blood loss (1.6 g/dL) as compared to the posterior group (2.5 g/dL) (*p* < .001) before propensity score matching. After matching, similar findings continued to be observed where lower blood loss is observed in the lateral group (1.5 g/dL) as compared to the posterior group (2.5 g/dL) (*p* < 0.001).

Blood transfusion requirements differed between the two groups. In the lateral approach group, 12 patients (14.8%) required blood transfusion, compared to 18 patients (32.1%) in the posterior approach group (*p* = 0.02) (Table 1). After propensity score matching, 8 patients (14.3%) in the lateral group required transfusion versus 18 patients (32.1%) in the posterior group (*p* = 0.03) (Table 2). The mean number of units transfused was also lower in the lateral group (1.2 ± 0.4 units) compared to the posterior group (1.8 ± 0.6 units) among those who required transfusion

**Table 1:** Patient demographics and outcome scores *before* propensity score matching. Unless otherwise stated, all results are median [IQR].

		Lateral	Posterior	*p*-Value
*n*		81	56	
Sex (%)	Female	49 (60.5)	39 (69.6)	.359
	Male	32 (39.5)	17 (30.4)	
Body mass index (kg/m^2^)		24.2 [22.2, 27.7]	27 [23.8, 31]	.007
Age (Years)		72 [64, 81]	66.1 [57.3, 72.6]	.001
Blood Loss (mean (SD))		1.6 (1.8)	2.5 (0.7)	.001
Blood transfusion requirement		12 (14.8)	18 (32.1)	.02
Cause for primary THR revision (%)	Aseptic loosening	25 (30.9)	32 (57.1)	<0.001
	Dislocation	7 (8.6)	15 (26.8)	
	Fracture	19 (23.5)	1 (1.8)	
	Infection	16 (19.8)	5 (8.9)	
	Others	14 (17.3)	3 (5.4)	
Revision THR complications (%)	Dislocation	1 (1.2)	9 (16.1)	.002
	Fracture	1 (1.2)	0 (0)	
	Infection	1 (1.2)	5 (8.9)	
	Nil	74 (91.4)	40 (71.4)	
	Others	4 (4.9)	2 (3.6)	
Revision THR dislocation (%)	No	80 (98.8)	47 (83.9)	.003
	Yes	1 (1.2)	9 (16.1)	
**Preoperative**				
Oxford (mean (SD))		40.7 (11.8)	38.6 (11.3)	.298
WOMAC	Pain	54 [32, 72]	72 [45.5, 90.5]	.042
	Stiffness	85 [50, 100]	87.5 [50, 100]	.871
	Physical function	40.6 [20.6, 63.5]	47.9 [27.1, 66.2]	.249
SF 36	Physical component score	27.7 [21.2, 31.7]	26.6 [20, 35.8]	.632
	Mental component score	54.2 [43.8, 63.1]	56.6 [45.9, 65.7]	.2
**Postoperative 6-month**				
Oxford		24.6 [18, 32.9]	21.5 [16, 28.2]	.08
WOMAC	Pain	100 [88, 100]	100 [91.5, 100]	.203
	Stiffness	95.7 [90, 100]	90 [73.8, 100]	.088
	Physical function	67.7 [50.2, 82.3]	73.5 [54, 86.5]	.255
SF 36	Physical component score	37.8 (10.1)	39.5 (12.1)	.389
	Mental component score	53.7 (11.6)	54.2 (11)	.792
Expectation Met (%)	Very Satisfied	29 (35.8)	27 (48.2)	.041
	Satisfied	39 (48.1)	14 (25)	
	Neutral	7 (8.6)	8 (14.3)	
	Dissatisfied	5 (6.2)	4 (7.1)	
	Very dissatisfied	1 (1.2)	3 (5.4)	
**Postoperative 2-year**				
Oxford		19.8 [17, 23.9]	21 [15, 26.3]	.464
WOMAC	Pain	100 [98.6, 100]	100 [90, 100]	.286
	Stiffness	93.8 [87, 100]	100 [90, 100]	.092
	Physical function	77.1 [68.1, 88.2]	79.3 [62.9, 91.8]	.951
SF 36	Physical component score	38.5 [33.1, 46.5]	43.7 [36, 51.1]	.173
	Mental component score	54.8 [48, 61.7]	53.6 [42.1, 62.9]	.641
Expectation Met (%)	Very satisfied	26 (32.1)	25 (44.7)	.008
	Satisfied	42 (51.9)	13 (23.2)	
	Neutral	12 (14.8)	12 (21.4)	
	Dissatisfied	0 (0)	5 (8.9)	
	Very dissatisfied	1 (1.2)	1 (1.8)	

**Table 2:** Patient demographics and outcome scores after propensity score. Unless otherwise stated, all results are median [IQR].

		Lateral	Posterior	p-value
n		56	56	
Sex (%)	Female	36 (64.3)	39 (69.6)	.688
	Male	20 (35.7)	17 (30.4)	
Body mass index (kg/m^2^)		24.4 [22, 28.2]	27 [23.8, 31]	.027
Age (Years)		70 [62, 79.2]	66.1 [57.3, 72.6]	.058
blood Loss (mean (SD))		1.5 (1.8)	2.5 (0.7)	<.001
Blood transfusion requirement		8 (14.3)	18 (32.1)	.003
Cause for primary THR revision (%)	aseptic loosening	24 (42.9)	32 (57.1)	.017
	dislocation	7 (12.5)	15 (26.8)	
	fracture	4 (7.1)	1 (1.8)	
	infection	10 (17.9)	5 (8.9)	
	Others	11 (19.6)	3 (5.4)	
Revision THR complications (%)	dislocation	1 (1.8)	9 (16.1)	.013
	fracture	0 (0)	0 (0)	
	infection	1 (1.8)	5 (8.9)	
	nil	50 (89.3)	40 (71.4)	
	others	4 (7.1)	2 (3.6)	
Revision THR dislocation (%)	No	55 (98.2)	47 (83.9)	.02
	Yes	1 (1.8)	9 (16.1)	
**Preoperative**				
Oxford (mean (SD))		40 (11.9)	38.6 (11.3)	.522
WOMAC	Pain	63 [35, 74]	72 [45.5, 90.5]	.106
	Stiffness	85 [50, 100]	87.5 [50, 100]	.983
	Physical function	41.5 [19.6, 63.5]	47.9 [27.1, 66.2]	.375
SF 36	Physical component score	28.3 [21.1, 32.2]	26.6 [20, 35.8]	.609
	Mental component score	55 [45.7, 63.9]	56.6 [45.9, 65.7]	.516
**Postoperative 6-month**				
Oxford		23 [18, 30.2]	21.5 [16, 28.2]	.233
WOMAC	Pain	100 [89.9, 100]	100 [91.5, 100]	.533
	Stiffness	95.4 [90, 100]	90 [73.8, 100]	.158
	Physical function	69.1 [53.4, 82.9]	73.5 [54, 86.5]	.526
SF 36	Physical component score	37.7 (9.2)	39.5 (12.1)	.379
	Mental component score	54.6 (11)	54.2 (11)	.841
Expectation Met (%)	Very Satisfied	21 (37.5)	27 (48.2)	.02
	Satisfied	28 (50)	14 (25)	
	Neutral	3 (5.4)	8 (14.3)	
	Dissatisfied	4 (7.1)	4 (7.1)	
	Very dissatisfied	0 (0)	3 (5.4)	
**Postoperative 2-year**				
Oxford		19.8 [16.8, 23.9]	21 [15, 26.3]	.463
WOMAC	Pain	100 [98.7, 100]	100 [90, 100]	.224
	Stiffness	93.6 [87, 100]	100 [90, 100]	.123
	Physical function	78 [68, 89.4]	79.3 [62.9, 91.8]	.903
SF 36	Physical component score	39.8 [35.3, 48.6]	43.7 [36, 51.1]	.436
	Mental component score	55.1 [49.2, 61.9]	53.6 [42.1, 62.9]	.333
Expectation Met (%)	Very satisfied	19 (33.9)	25 (44.7)	.019
	Satisfied	30 (53.6)	13 (23.2)	
	Neutral	7 (12.5)	12 (21.4)	
	Dissatisfied	0 (0)	6 (10.7)	
	Very dissatisfied	0 (0)	0 (0)	

### Functional outcomes

Prior to matching, there was noticed difference between the WOMAC pain score between the lateral and posterior group (54 vs. 72, *p* = 0.042), while there was no difference for WOMAC stiffness, physical function, Oxford score, and SF 36 scores. After matching, the earlier difference between lateral and posterior groups for the WOMAC pain score was no longer observed (Table 2). And there was no significant difference noted between the two groups for WOMAC stiffness, physical function, Oxford score, and SF 36 scores at 6 months and 2 years post-operatively.

### Satisfactions scores

After propensity score matching at six months post-operatively, a larger proportion of patients expressed being satisfied or very satisfied (*n* = 49, 87.5%) in the lateral group as compared to the posterior group (*n* = 41, 73.2%). This phenomenon continued at two years post-operatively, where forty-nine patients (87.5%) were satisfied or very satisfied with their outcomes as compared to the posterior group, where only thirty-eight patients were satisfied or very satisfied (67.9%).

### Radiological measurements

Radiological analysis for cup placement was done for all 137 patients by three senior surgeons with the average measurement taken. Measurements were done using a computerized, calibrated scale calculator via the PACS system. Acetabular cup abduction is measured on radiographs, taking reference to Lewinnek’s safe zone, which suggested an abduction angle of 40 degrees ± 10 degrees. In our series of patients, 82% of cup placement via the lateral approach and 78% of cup placement via the posterior approach were within the Lewinnek safe zone. There was no statistical significance between the two.

## Discussion

### Dislocations in revision THR

Dislocation is a leading cause of failure and need for re-revision after revision THR [13], with epidemiological studies reporting dislocation rates as high as 22.5% in revision THR [14]. While dislocation rates for the posterior approach in primary THR range from 1% to 5% [15] and lower dislocation rates for the lateral approach primary THR, ranging from 0.4% to 0.55% [9, 16]. Our analysis showed that patients undergoing revision THR via the lateral approach have a significantly lower dislocation rate compared to the posterior group (1.8% vs. 16.1%, Table 2), with dislocation rates similar to those reported in a primary THR. In a large cohort study of 1211 revision total hip replacements, Wetters et al. reported a dislocation rate of approximately 9.8% (*n* = 113) in revision hip surgery utilizing the posterior approach [17]. However, dislocation rates comparing lateral and posterior approaches in revision THRs are scarce, which makes it difficult for surgeons to decide on the choice of approach in revision THRs. To our knowledge, this study is one of two studies to observe and report on dislocation rates comparing the lateral and posterior approaches in revision total hip replacements (Table 3).

**Table 3:** Comparative studies of lateral versus posterior approaches in revision total hip replacement.

Study (Author, Year)	Study type	Total *N* (lateral/posterior)	Dislocation Rate (%)	Blood loss	Functional outcomes	Follow-up duration	Key findings
			Lateral vs. Posterior				
Robertson et al, 2022	Retrospective cohort	*N* = 248 (80/114)*	6% vs. 14% (*p* = 0.13, NS)	*NR*	*NR*	Mean 2.3 years	No significant difference in dislocation rates between approaches. Head/liner exchange only.
Morley et al, 2017	Retrospective cohort	*N* = 209 (43/166)**	*NR by revision approach*	*NR*	Posterior groups: better Oxford (*p* = 0.028) and WOMAC (*p* = 0.026)	Mean 5 years	Posterior approach associated with better PROMs. Grouped by approach concordance, not pure lateral vs. posterior.
Harmer et al, 2022	Retrospective cohort	*N* = 705 (approach concordance study)***	No difference concordant vs. discordant approaches	*NR*	*NR*	Mean 4 years	Changing surgical approach from primary to revision not associated with increased complications. Focus on concordance, not specific approach comparison.
Current study	Retrospective cohort with PSM	*N* = 137 (81/56); PSM: (56/56)	1.8% vs. 16.1% (*p* = 0.02)	1.5 vs. 2.5 g/dL (*p* < 0.001)	No difference in Oxford, WOMAC, SF-36 at 6mo and 2 yr. Higher satisfaction: 87.5% vs. 67.9% at 2yr (*p* = 0.019)	Min 2 years	Significantly lower dislocation and blood loss with lateral approach. First study with comprehensive head-to-head comparison in revision THR.

NR = Not Reported; NS = Not Significant; PSM = Propensity Score Matching; PROMs = Patient-Reported Outcome Measures.

*Robertson 2022: Also included 54 direct anterior revisions (17% dislocation). Study specific to head and liner exchange only.

**Morley 2017: Groups were Lateral/Lateral (n = 43), Lateral/Posterior (n = 43), Posterior/Posterior (n = 123). Study grouped by approach concordance rather than pure lateral vs. posterior comparison in revision.

***Harmer 2022: Examined whether changing approaches matters (concordant vs. discordant). Did not provide specific lateral vs. posterior dislocation data.

Recent work by Wyles and colleagues has emphasized the importance of patient-specific risk stratification for dislocation following total hip arthroplasty. Their comprehensive analysis of nearly 30,000 procedures identified multiple non-modifiable risk factors, including advanced age, female sex, elevated body mass index, underlying diagnosis (particularly fracture and avascular necrosis), presence of spine disease, prior spine surgery, and neurologic conditions [18]. These factors, when combined with surgical approach and implant choices, can significantly influence instability risk [19]. In our revision cohort, we observed favorable outcomes with the lateral approach, but surgeons should consider these patient-specific factors when selecting the optimal surgical approach and implant configuration for each individual patient.

### Blood loss

Patients undergoing revision hip surgeries are known to be at greater risk of intraoperative blood loss, which may require blood transfusions [20]. Blood loss during revision hip surgeries has been shown to be significantly higher in men, older patients, and those undergoing revision of dual components or cemented implants [21]. In our study, there was significantly lower blood loss in the lateral group as compared to the posterior groups (1.5 g/dL vs. 2.5 g/dL, *p* < 0.001, Table 2). The authors believe that the Hardinge approach in a revision setting contributes to lower blood loss, as it allows for better soft tissue dissection and visualization in untainted tissue planes. In contrast, the posterior approach required significant dissection through scar tissue, making visualization and dissection more difficult, leading to increased bleeding.

### Dual mobility

The advent of dual mobility acetabular components has provided surgeons with an additional tool to address instability in revision total hip arthroplasty. A systematic review and meta-analysis by Placella et al*.* found a lower dislocation risk with dual-mobility than fixed-bearing cups in revision THR [22]. These implants have been particularly adopted in conjunction with the posterior approach to mitigate the higher instability risk associated with this approach. While dual mobility components offer biomechanical advantages in terms of jump distance and increased range of motion before impingement, they are not without potential complications, including intraprosthetic dislocation and higher costs [22]. In countries where dual mobility constructs have become standard practice for high-risk revision cases, particularly those using the posterior approach, surgeons have observed improved stability outcomes. Moreover, improved techniques of dual mobility using cemented DM cup in a CM acetabular component as described by Blum et al. [23] have shown favourable outcomes, which are likely to further increase the usage of dual mobility implants in revision total hip replacements.

### Radiological analysis

Reasons for unstable total hip replacements leading to revision surgery have been categorised into six categories: acetabular component malposition 33%, femoral component malposition 8%, abductor deficiency 36%, impingement 9%, late wear 7%, and idiopathic 7% [24]. Acetabular component malposition is a significant factor in recurrent dislocation. A major guideline of component positioning was described by Lewinnek in his landmark paper of Safe Zones, which is 40 ± 10 degrees [25]. While we acknowledge that recent studies have questioned the utility of Lewinnek safe zones specifically for dislocation risks, we feel that it provides an easily understood reference point for comparison. In our study, the majority of cup placements in lateral approach and posterior approach revision THRs fall within the safe zone (82%, compared to 78%) with no statistical significance. Representative radiographic examples from our cohort demonstrate typical cup positioning achieved through both approaches, though detailed radiographic analysis was beyond the scope of this registry study.

### Disadvantages of lateral approach

While our study demonstrates favorable outcomes with the lateral approach in revision THR, it is important to acknowledge the potential downsides of this technique. The direct lateral approach carries inherent risks, including damage to the gluteus medius and minimus muscles, which may result in persistent abductor weakness and Trendelenburg gait [8]. Superior gluteal nerve injury, though relatively uncommon, represents a serious complication that can lead to permanent abductor dysfunction [26]. Additionally, the lateral approach has been associated with a higher incidence of heterotopic ossification compared to other approaches, which may impact functional outcomes in some patients. The requirement for careful repair of the abductor mechanism and potential for incomplete healing add technical demands to the procedure. These considerations underscore the importance of careful patient selection, meticulous surgical technique with proper tissue handling, and appropriate rehabilitation protocols when utilizing the lateral approach for revision THR.

### Strengths and limitations of the study

This study was conducted at a single tertiary center with a demographic distribution similar to that of the local population. All surgeries were performed by fellowship-trained arthroplasty surgeons. There are several articles within the literature that discuss the optimal surgical approach in revision total hip replacements [27, 28]. Discussion on choice of approach is influenced by several factors, such as the indication for revision, previous choice of implant and fixation type, the state of soft tissue and bone, previous incisions used, patient factors, the surgeon's experience and training [28, 29]. Numerous studies have been published in the literature comparing the lateral and posterior approach in primary hip surgeries [8, 9], there is a paucity with regard to approaches in revision hip surgeries. To our knowledge, this is the first study comparing the outcomes of surgical approaches in revision settings with regard to complications and functional outcomes. Our study’s limitations include its relatively small sample size, single-center design, and retrospective nature. The lack of systematic documentation of transfusion requirements and detailed radiographic analysis, including representative images, represents additional limitations that should be addressed in future prospective studies.

### Causes and complications in revision THR

Aseptic loosening, instability, and periprosthetic joint infection are the most common reasons for revision THR [4, 6]. With the increasing lifespan of hip replacements and patients' activity level, the incidence of revision THRs is also rising, presenting a greater challenge for arthroplasty surgeons. Furthermore, the rise in chronic diseases, such as diabetes mellitus, chronic kidney diseases, hypertension, and increasingly, patients with solid organ transplants, among others, poses challenges for both the surgeon and anaesthetist in revision settings. Revision THR itself has a high re-revision rate of 15.8% [29]. Possible complications that can arise in the revision setting include infection, dislocation, venous thromboembolism, re-revision surgery, and mortality [29]. Our study's observations support the current literature, which identifies aseptic loosening as the most common cause for revision surgery (41.6%, *n* = 57, Table 1) and overall dislocation rates of 7.3% after revision THRs (*n* = 10, Table 1).

## Conclusion

Lateral approach in revision total hip replacement is a viable option with favorable outcomes, given its significantly lower dislocation rates and blood loss compared to the posterior approach. However, both approaches have their merits and limitations, and the choice should be individualized based on patient-specific risk factors, surgeon experience, and clinical circumstances. The potential for abductor damage with the lateral approach and the higher dislocation risk with the posterior approach (which may be mitigated with dual mobility constructs) should be carefully considered. We call for further studies to confirm our findings and evaluate the effects of surgical approaches on outcomes in revision hip surgery.

## Data Availability

Data will be available upon request.
